# Supplementary material for: Assessing the fitness of a dual-antiviral drug resistant human influenza virus in the ferret model
Source: Commun Biol. 2022 Sep 28;5:1026. doi: 10.1038/s42003-022-04005-4 (PMC9517990; doi:10.1038/s42003-022-04005-4)
Supplement: Supplementary file 2 — Supplementary information [file 42003_2022_4005_MOESM2_ESM.pdf]

Supporting information

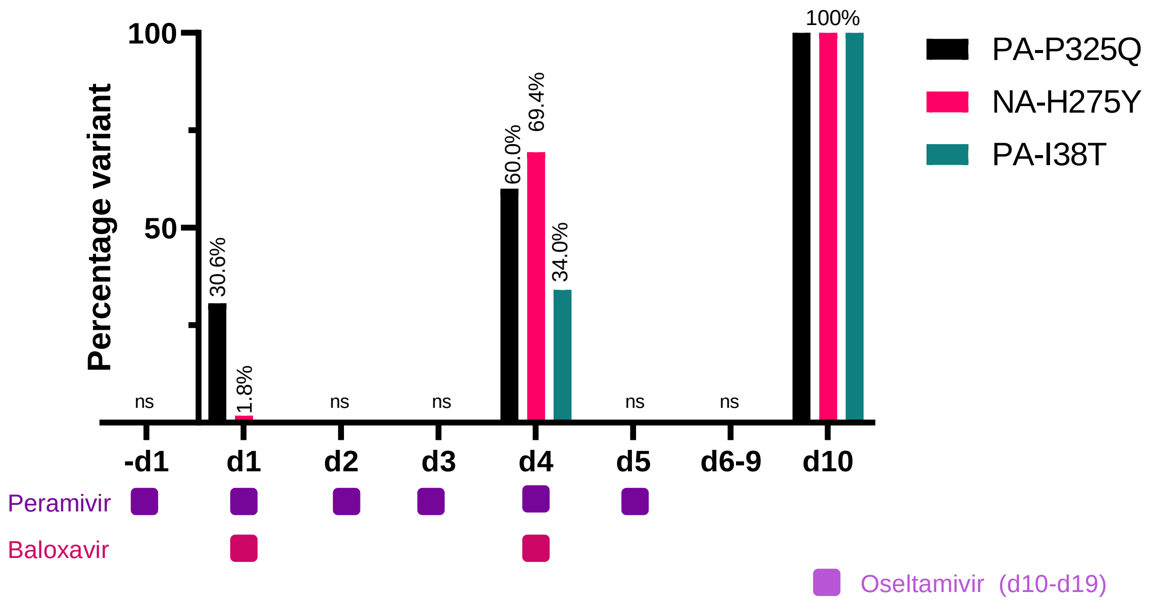

Supplementary Figure 1. The timeline of the patient's antiviral treatment and proportions variants as in the respiratory clinical samples determined by whole-genome sequencing. The treatment and viral isolate genotype time course of a 71-year-old female receiving palbociclib for metastatic breast cancer with severe influenza. Baloxavir marboxil (Baloxavir) was administered orally at 40 mg at day 1 (d1) and d4 of enrolment. Peramivir was administered daily by intravenous route at 400 mg/mL on one day prior to enrolment (-d1) and d1-5. Oseltamivir was administered orally at 30 mg twice-daily d10-19. Percentage values indicate the proportion of PA-P325Q (black), NA-H275Y (red) and PA-I38T (green) substitutions detected by iSeq next-generation sequencing on the clinical samples. No sequence available where indicated, ns.

Supplementary Table 1. Whole genome sequenced results of clinical samples and isolated viruses, illustrating amino acid changes compared to the virus consensus genome from the patient's day 1 post enrolment sample("-", represents no detected changes).

**Clinical samples**								
Clinical sample Name	***PB2***	***PB1***	***PA***	***HA***	***NP***	***NA***	***M***	***NS***
Day 1 of enrolment	-	-	0%<br>I38T<br>30.6%<br>P325Q	-	-	1.8%<br>H275Y	-	-
Day 4 of enrolment	-	-	34%<br>I38T<br>60%<br>P325Q	-	-	69.4%<br>H275Y	-	-
Day 10 of enrolment	-	-	100%<br>I38T<br>100%<br>P325Q	-	-	100%<br>H275Y	-	-
**Clinically isolated and plaque purified virus isolates**								
Isolate Name	***PB2***	***PB1***	***PA***	***HA***	***NP***	***NA***	***M***	***NS***
A/South Korea/90207_<br>d1_A/2020<br>(Day 1; WT)<br>GISAID ID: 13655148	-	100%<br>Y129H	0%<br>I38T<br>0%<br>P325Q	100%<br>A204T	-	0%<br>H275Y-	-	-
A/South Korea/90207_<br>d1_B/2020<br>(Day 1; NA-H275Y)	-	-	0%<br>I38T<br>0%<br>P325Q	-	-	100%<br>H275Y	-	-
A/South Korea/90207_<br>d4_C/2020<br>(Day 4; PA-I38T)	-	-	100%<br>I38T<br>100%<br>P325Q	-	-	100%<br>S442I	-	100%<br>N209D
A/South Korea/90207_<br>d10_D/2020<br>(Day 10; NA-H275Y+PA-I38T)<br>GISAID ID: 13655147	-	-	100%<br>I38T<br>100%<br>P325Q	--	-	100%<br>H275Y	-	-

Supplementary Table 2. Phenotypic antiviral susceptibility results revealing the IC_{50} nM ± SD (fold change relative to WT) of each clinical virus isolates (NCT-03684044) to four NAI antiviral drugs (n=3); oseltamivir (active compound oseltamivir carboxylate), peramivir, laninamivir, zanamivir, and a polymerase inhibitor antiviral drug baloxavir marboxil, active compound baloxavir acid (n=6).

**Mutations in A/South Korea/ 90207/2020 virus**	**IC_{50}nM ± SD (Fold change compared to WT)**				
	**Oseltamivir**	**Peramivir**	**Laninamivir**	**Zanamivir**	**Baloxavir***
**WT**	0.33 ± 0.10	0.16 ± 0.04	0.52 ± 0.16	0.38 ± 0.10	1.0 ± 0.2
**NA-H275Y**	190.7 ± 41.2 **(602)**	16.8 ± 3.1 **(106)**	1.16 ± 0.15 **(2)**	0.43 ± 0.12 **(1)**	1.2 ± 0.3 **(1)**
**PA-I38T**	0.54 ± 0.13 **(2)**	0.26 ± 0.04 **(2)**	1.26 ± 0.02 **(3)**	0.63 ± 0.16 **(2)**	379.7 ± 120.6 **(373)**
**NA-H275Y +PA-I38T**	205.6 ± 33.7 **(659)**	16.2 ± 2.94 **(103)**	1.10 ± 0.36 **(2)**	0.46 ± 0.09 **(1)**	193.8 ± 28.1 **(190)**

*Baloxavir phenotypic cell based assay differs to the NAI enzymatic assay, and baloxavir assay readout was measured as half-maximal effective concentration (EC_{50}).

Supplementary Table 3. In vitro replication of WT, NA-H275Y, PA-I38T and NA-H275Y+PA-I38T clinical virus isolates pyrosequencing analysis of supernatant at gene position NA-275 and PA-38 at 102 hours post infection.

**Inoculum**	**Exp. repeat #**	**Mixed base at NA-275**	**Mixed base at PA-38**	**Inoculum**	**Exp. repeat #**	**Mixed base at NA-275**	**Mixed base at PA-38**
**WT**	1	99.6% H	100% I	**PA-I38T**	1	99.9% H	92.5% T
		96.5% H	100% I			95.1% H	71.5% T
		100% H	100% I			98.9% H	98.4% T
	2	98.7% H	100% I		2	95.3% H	89.6% T
		100% H	100% I			96.1% H	96.3% T
		100% H	100% I			99% H	100% T
	3	100% H	100% I		3	96.7% H	97% T
		99.9% H	100% I			100% H	100% T
		100% H	100% I			100% H	95.1% T
**NA-H275Y**	1	97.6% Y	100% I	**NA-H275Y +PA-I38T**	1	98.5% Y	90.8% T
		97.3% Y	100% I			98.3% Y	93.8% T
		100% Y	100% I			97% Y	97.1% T
	2	98.1% Y	100% I		2	98.7% Y	94% T
		96.7% Y	100% I			98% Y	96.5% T
		100% Y	100% I			100% Y	94.8% T
	3	100% Y	100% T		3	100% Y	100% T
		100% Y	100% T			100% Y	100% T
		100% Y	100% T			100% Y	100% T

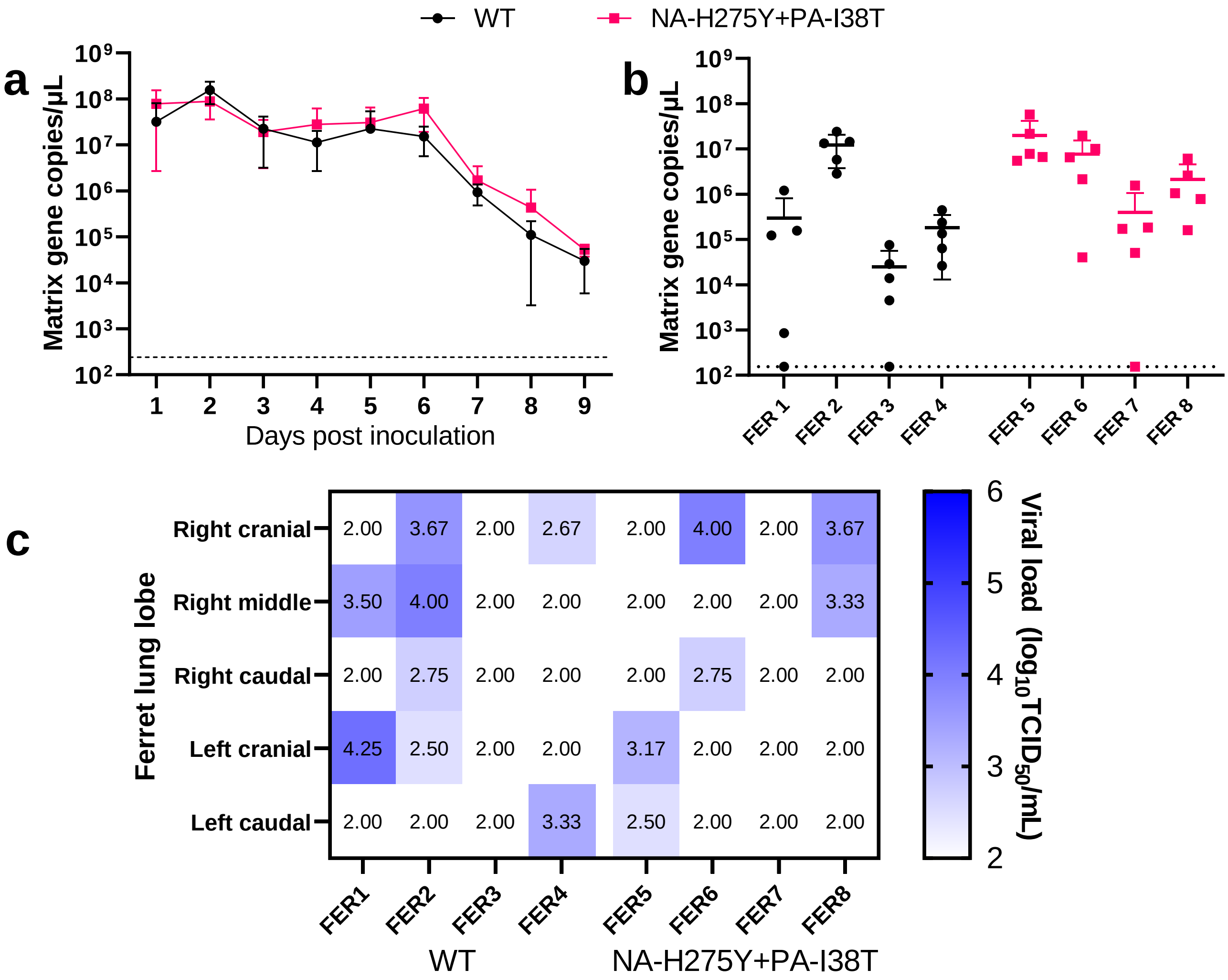

Supplementary Figure 2. Viral replication *in vivo* study, viral shedding and lung lobe titre of WT and NA-H275Y+PA-I38T virus isolate infected ferrets. Ferrets (n=8) infected by the intranasal route with 4 $\log_{10}$TCID$_{50}$/mL (in 500 µL of PBS) with WT (d1 isolate, black) or NA-H275Y+PA-I38T (d10 isolate, red) purified virus isolates were nasally washed daily for 9 days. Half of the ferrets that were culled at day five post infection to harvest the five major lung lobes. (a) The viral shedding from the ferret nasal washes was measured by qPCR for influenza A matrix gene amplification. No statistically significant ($p<0.05$) differences were observed (Two-way ANOVA with Sidak's multiple comparison). (b) Viral titres of each lung lobe supernatant aliquot (homogenised individually in 5 mL of PBS) were enumerated by qPCR. Each dot represents the titre of a single lung lobe and the line shows the mean. Error bars represent one SD. LOD is shown as a dotted horizontal line. (c) Heat map illustrates the infectious viral titre for each specific lung lobe from white, 2 $\log_{10}$TCID$_{50}$/mL (LOD), to blue, 6 $\log_{10}$TCID$_{50}$/mL. All values below LOD equal 2 $\log_{10}$TCID$_{50}$/mL.

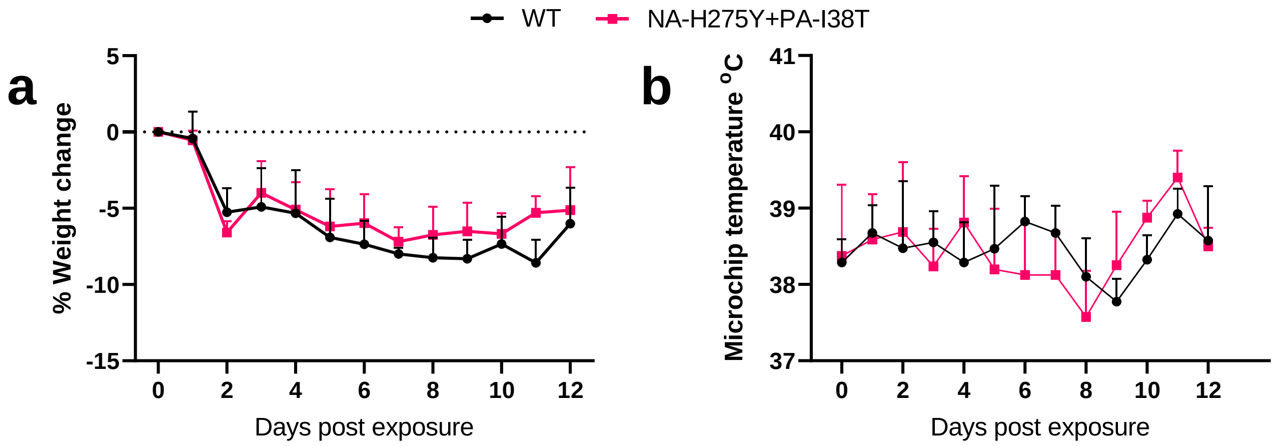

Supplementary Figure 3. Influenza A(H1N1)pmd09 NA-H275Y+PA-I38T viral replication *in vivo* study, ferret weight loss and body temperature over duration of experiment. Graphs illustrate the weight loss for WT (black) and NA-H275Y+PA-I38T (red) inoculated ferrets over the duration of infection (n=8 D1-D5, n=4 D6-D12). (a) Weight loss line graph compares the mean percentage of the change in weight from pre-experimental weight between the two virus groups. (b) Body temperature of ferrets measured daily by subcutaneous microchip readout. Error bars indicate one SD. Two-way ANOVA Sidak's multiple comparison test analysis was performed on each time point of weight loss and temperature, no significant difference ($p<0.05$) was observed.

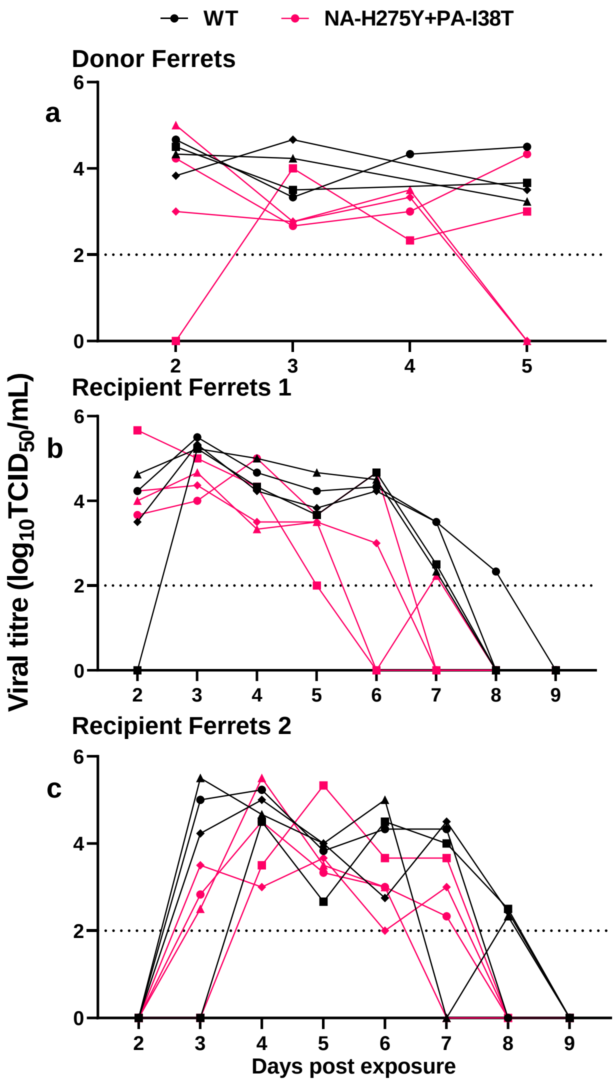

Supplementary Figure 4. Individual infectious viral shedding in nasal washes from Influenza A(H1N1)pmd09 WT or NA-H275Y+PA-I38T clinical isolate infected donors and airborne transmission infected recipients. Donor ferrets (n=4) were infected by the intranasal route with 5 $log_{10}TCID_{50}$/mL of pure WT or pure NA-H275Y+PA-I38T clinically isolated virus and co-housed with naïve recipient ferret 1 (RF1) separated by an airborne virus-permeable barrier at day 1 (D1) post inoculation. Infected RF1s were then co-housed with RF2 ferrets, enabling a second airborne transmission event to occur. Viral titre from each nasal washes from donors (a), RF1 (b) and RF2 ferrets (c), by $TCID_{50}$ assay. LOD is shown as a dotted horizontal line, all values below LOD equal zero.

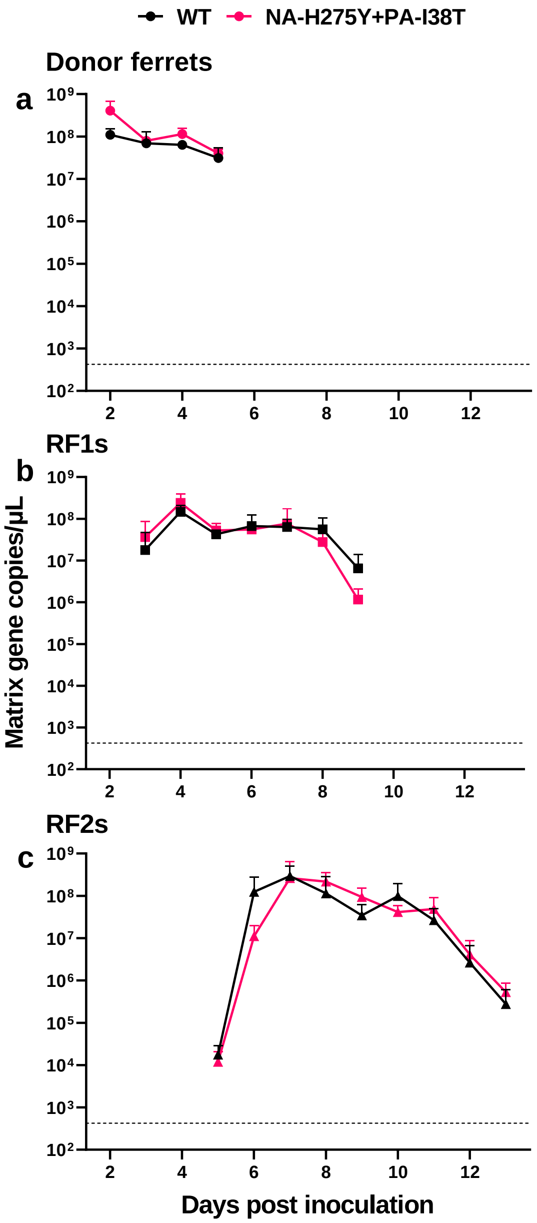

Supplementary Figure 5. Viral RNA shedding in nasal washes from ferrets infected with WT or NA-H275Y+PA-I38T clinical isolates and in ferrets infected by airborne virus exposure. Donor ferrets (n=4) were infected by the intranasal route with 5 $\log_{10}TCID_{50}$/mL of pure WT or pure NA-H275Y+PA-I38T clinically isolated virus and co-housed with naïve recipient ferret 1 (RF1) separated by an airborne virus-permeable barrier at day 1 (D1) post inoculation. Infected RF1s were then co-housed with RF2 ferrets, enabling a second airborne transmission event to occur. Viral RNA load of each ferret nasal wash was measured by qPCR analysis, with the mean matrix gene copies per µL of RNA from WT (black) and NA-H275Y+PA-I38T (red) infected donor ferrets (a), RF1s (b) and RF2s (c) over time (days post inoculation of donor). Error bars indicate one SD. No significant differences ($p<0.05$) were observed, analysed by Two-way ANOVA, Sidak's multiple comparison. LOD is shown as a dotted horizontal line.

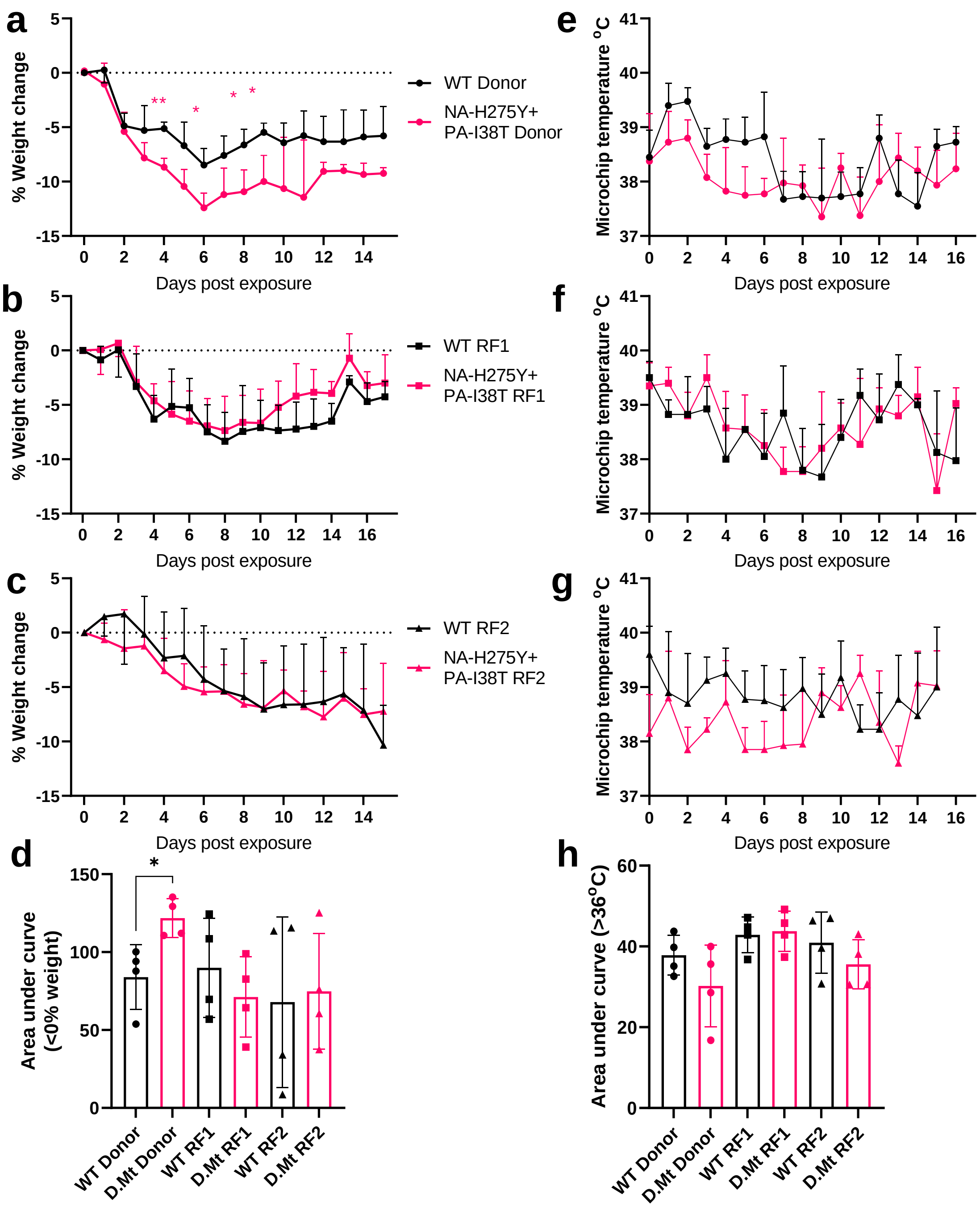

Supplementary Figure 6. Influenza A(H1N1)pmd09 NA-H275Y+PA-I38T airborne transmission study, ferret weight loss and body temperature over duration of experiment, with area under curve analysis. The percentage change from pre-experimental weight for (a) donor, (b) recipient ferret 1 (RF1) and (c) RF2 ferrets recorded daily over the duration of exposure to infectious virus is illustrated in the left panel of line graphs. Daily body temperature readouts from sub-cutaneous microchip are depicted by line graph for (e) donor, (f) RF1 and (g) RF2 ferrets. Each line graph compares WT (black) with double mutant (D.Mt) NA-H275Y+PA-I38T (red) virus

inoculated/exposed animals. Two-way ANOVA Dunnett's multiple comparison test illustrate the significant difference (p<0.05) between each WT and NA-H275Y+PA-I38T (red asterisks) infected ferrets. The bar graphs depict the area under the curve of % weight change (d) and temperature (h), calculated for each individual ferret (n=4), and compared between groups of ferrets by One-way ANOVA Tukey's multiple comparison test analysis (asterisk for p<0.05). Error bars indicate one SD. Note that: one NA-H275Y+PA-I38T infected donor was euthanised at D11 due to surpassing humane weight loss threshold of 15%, therefore data points from D12-D15 were the mean of three ferrets.

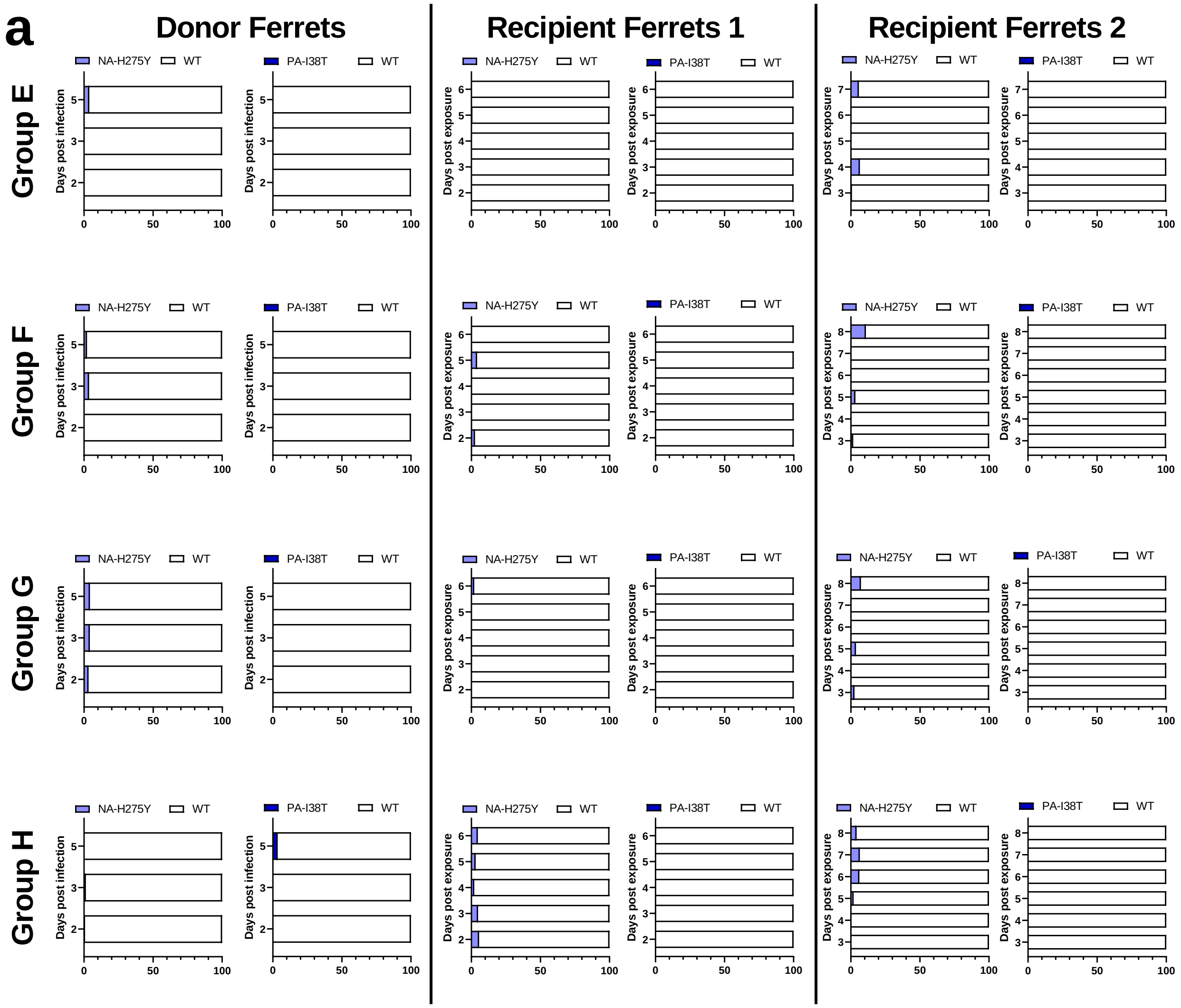

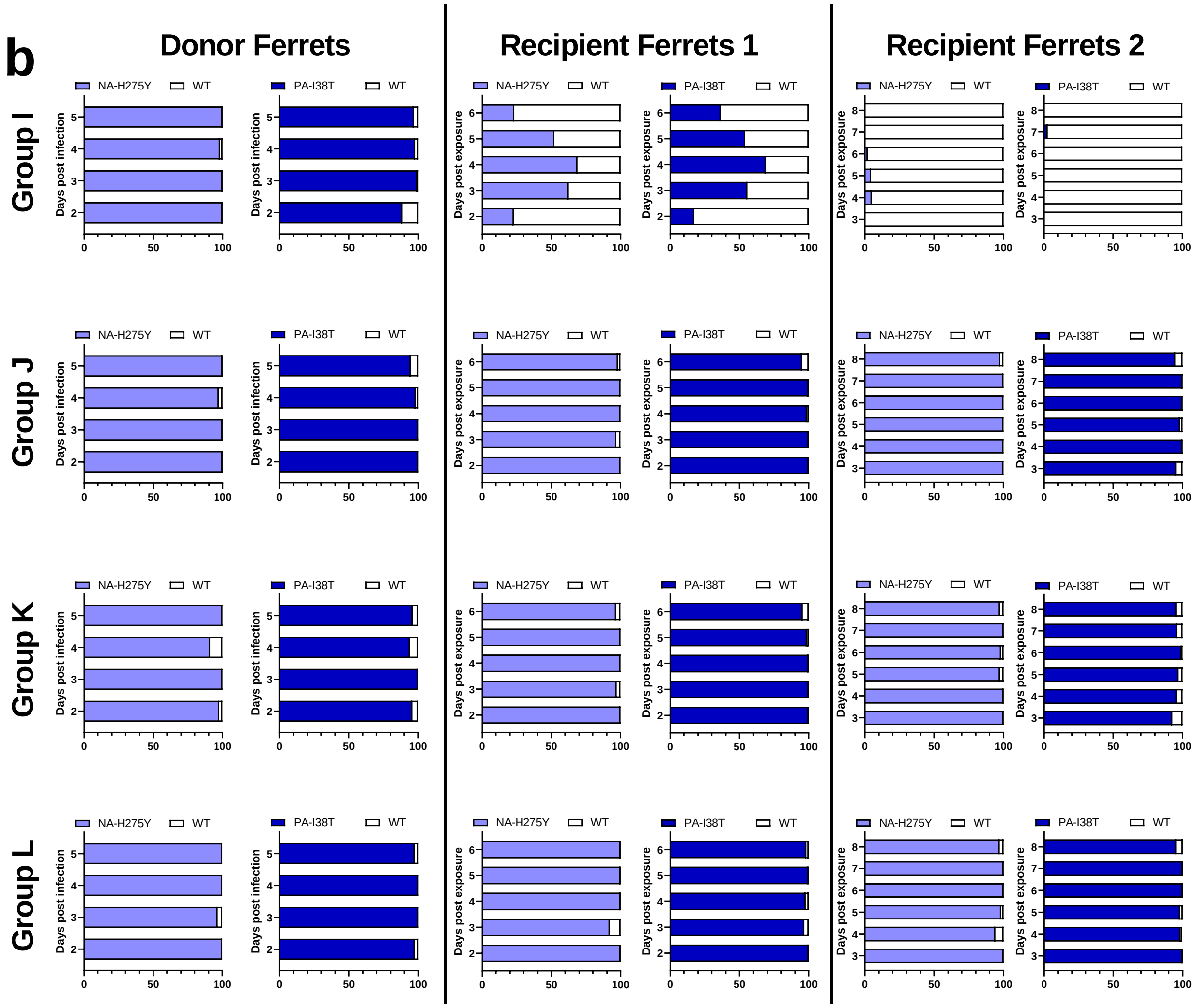

Supplementary Figure 7. Pyrosequencing analysis of ferret nasal washes from WT clinical isolate inoculated donor and airborne transmission infected recipient ferrets. As previously outlined, donor ferrets (n=4) were infected by the intranasal route with 5 $log_{10}TCID_{50}/mL$ with pure (a) WT or (b) NA-H275Y+PA-I38T clinical isolate and co-housed with naïve recipient ferret 1 (RF1) separated by an airborne virus-permeable barrier, infected RF1s were then co-housed with RF2 ferrets. Pyrosequencing analysis displayed the relative proportion of virus encoding WT PA-I38 (white bars) and PA-I38T (dark blue bars) as well as WT NA-H275 (white bars) and NA-H275Y (light blue bars) for each ferret nasal wash with detectable virus (>2 $log_{10}TCID_{50}/mL$). Results are shown as columns of paired bar charts for each donor, RF1 and RF2 ferret, and each group (donor-RF1-RF2 transmission pairs) is shown in rows.

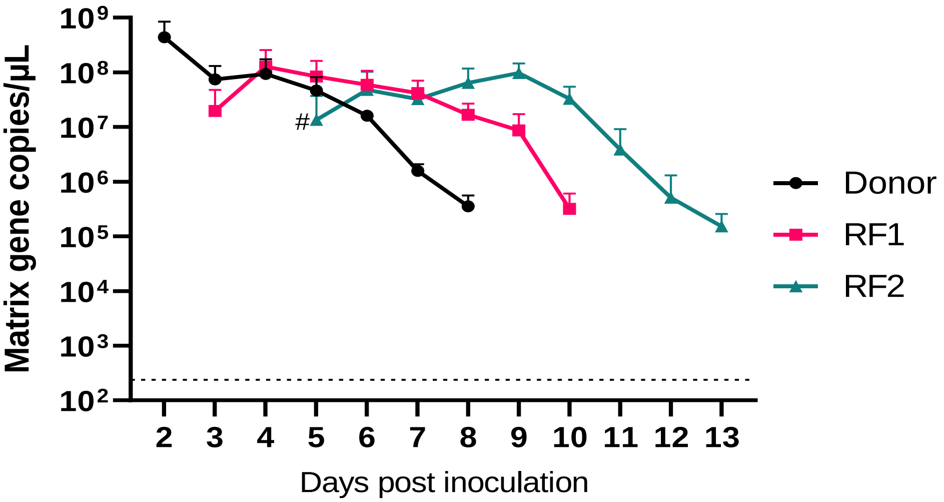

Supplementary Figure 8. Viral load of nasal washes from competitive ferret airborne transmission study of 50:50 WT: NA-H275Y+PA-I38T virus infected donor and airborne transmission infected recipient ferrets. Donor ferrets (n=4) were inoculated by the intranasal route with 5 $log_{10}TCID_{50}$/mL of 50:50 WT: NA-H275Y+PA-I38T virus and co-housed with naïve recipient ferret 1 (RF1) separated by permeable barrier at day 1 (D1) post inoculation. Infected RF1s were then co-housed with RF2 ferrets. Viral RNA load of each donor ferret nasal wash, with the mean matrix gene copies per µL of RNA from 4 ferret nasal wash samples of donor (black), RF1 (red) and RF2 (green) infected animals analysed by qPCR. "#" one of four RF2s was not nasally washed this day, as it was exposed one day later, given its RF1 transmission pair became virus positive on D4, not D3 like the other three RF1 animals. Error bars indicate one SD and the LOD is shown as a dotted horizontal line.

Supplementary Table 4. Whole genome sequencing on ferret inoculum and representative nasal wash samples from WT, NA-H275Y+PA-I38T and 50% WT: 50% NA-H275Y+PA-I38T virus infected ferrets post inoculation/exposure (PI/E).

Supplementary Table 3 Footnote: Clinical isolate, prepared ferret inoculum and representative nasal wash samples were extracted for viral RNA and amplified by PCR for library preparation and Illumina i-Seq analysis. Amino acid (AA) changes are listed in the table below across HA, NA, PA and PB1 genes where AA changes were present between the clinical isolate WT and NA-H275Y+PA-I38T viruses. Percentage frequency of AA changes is determined from variant bases called with a read depth over 1000 and frequency greater than 1%.

Sample		Gene			
		HA	NA	PA	PB1
**Clinical Isolates (pre-plaque purification and expansion)**					
**D1 WT**		100% A204	1.8% H275Y	100% I38<br>30.6% P325Q	100% Y129
**D10 NA-H275Y+PA-I38T**		100% A204	100% H275Y	100% I38T<br>100% P325Q	100% Y129
**Ferret inoculum (post-plaque purification and expansion)**					
**WT**		100% A204T	100% H275	100% I38<br>100% P325	100% Y129H
**NA-H275Y+PA-I38T**		100% A204	100% H275Y	100% I38T<br>100% P325Q	100% Y129
**50:50 WT:NA-H275Y+PA-I38T**		68% A204T	41% H275Y	31% I38T<br>28% P325Q	67% Y129H
**Ferret nasal washes 50:50 competitive mix infected ferrets**					
**Donor Ferrets D2 PI**	**Group A**	46% A204T	50% H275Y	37% I38T<br>39% P325Q	50% Y129H
	**Group B**	61% A204T	42% H275Y	44% I38T<br>39% P325Q	57% Y129H
	**Group C**	48% A204T	49% H275Y	42% I38T<br>40% P325Q	52% Y129H
	**Group D**	59% A204T	43% H275Y	39% I38T<br>39% P325Q	56% Y129H
**RF1 D2 PE**	**Group B**	100% A204	100% H275	100% I38<br>100% P325	100% Y129
	**Group C**	71% A204T<br>29% V544I	36% H275Y	87% I38T<br>87% P325Q	80% Y129H
**RF1 D5 PE**	**Group C**	90% A204T<br>11% V544I	100% H275	73% I38T<br>72% P325Q	34% Y129H
**RF2 D5 PE**	**Group A**	100% A204T	100% H275	13% I38T<br>12% P325Q	100% Y129
	**Group B**	100% A204T	100% H275	100% I38<br>100% P325	100% Y129
	**Group C**	100% A204	100% H275	100% I38T<br>100% P325Q	100% Y129
	**Group D**	91% A204T	100% H275	100%I38<br>100%P325	Undetermined
**Ferret nasal washes WT infected ferrets**					
**Donor D2 PI**	**Group G**	100% A204T	100% H275	100%I38<br>100% P325	100% Y129H
**RF1 D3 PE**	**Group G**	100% A204T	100% H275	100% I38<br>100% P325	100% Y129H
**RF2 D5 PE**	**Group G**	100% A204T	100% H275	100% I38<br>100% P325	100% Y129H
**Ferret nasal washes NA-H275Y+PA-I38T (D.Mt) infected ferrets**					

**Donor D2 PI**	**Group I**	100% A204	100% H275Y	100% I38T 100% P325Q	100% Y129
	**Group L**	100% A204	100% H275Y	100% I38T 100% P325Q	100% Y129
**RF1 D3 PE**	**Group I**	36% A204T	64% H275Y	69% I38T 56% P325Q	39% Y129H
	**Group L**	100% A204	100% H275Y	100% I38T 100% P325Q	100% Y129
**RF1 D6 PE**	**Group I**	30% A204T	26% H275Y	63% I38T 34% P325Q	17% Y129H
**RF2 D5 PE**	**Group I**	100% A204	100% H275	100% I38 100% P325	100% Y129
	**Group L**	100% A204	100% H275Y	100% I38T 100% P325Q	100% Y129

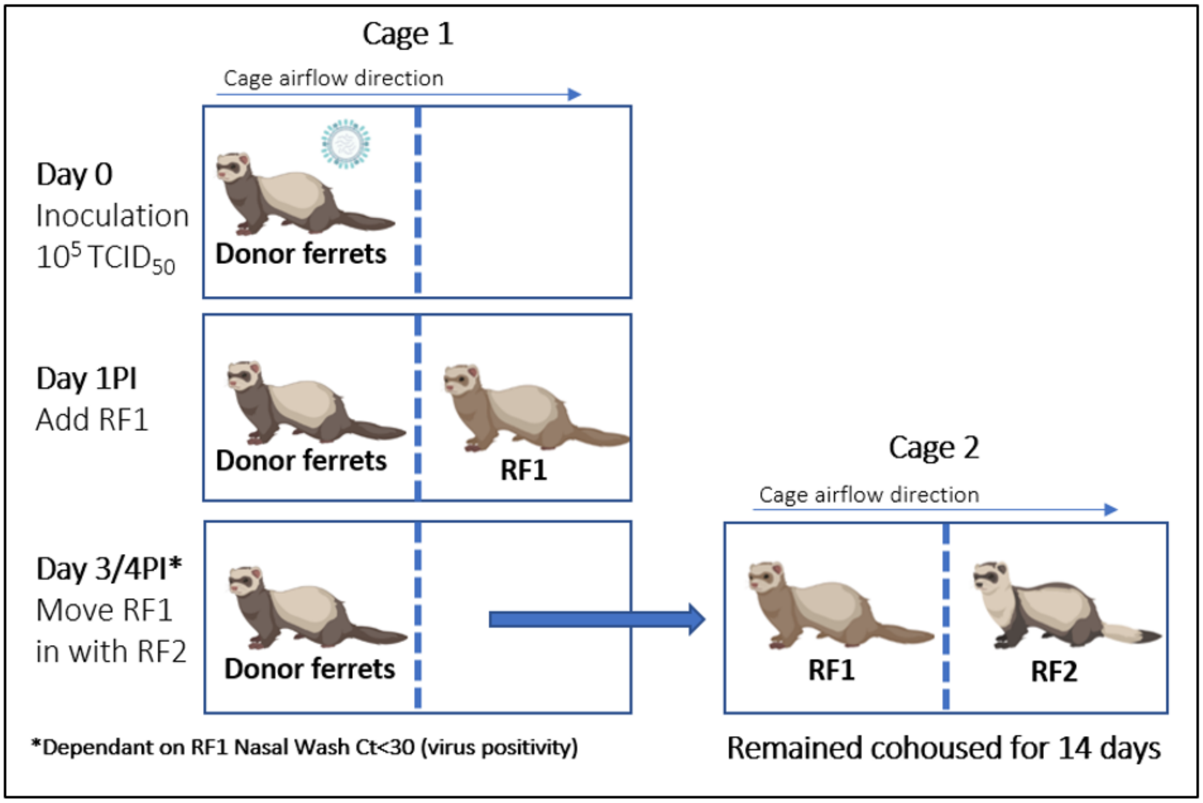

Supplementary Figure 9. Transmission chain of ferret airborne transmission. Inoculated donor ferrets were added to the cage with naïve recipient ferret 1 (RF1) one day post infection. Animals were separated with a perforated metal sheet (represented by dotted line) in cages aerated with one-directional airflow. The day RF1 was virus positive (as determined by nasal wash qPCR, Ct <30), RF1 was moved to the cage adjacent to RF2, where the animals were co-housed for the remainder of the trial (14 days).

Supplementary Table 5. Primers for pyrosequencing analysis and predicted sequence

Target	Influenza A(H1N1)pdm09 PA/I38T (5’ to 3’)	Influenza A(H1N1)pdm09 NA/H275Y (5’ to 3’)
**RT-PCR Forward Primer**	Biotin-CAATCCAATGATCGTCGAGC	GACAGGCCTCATACAAGATCTTC
**RT-PCR Reverse Primer**	GGTGCTTCAATAGTGCATTTGG	Biotin-TGCCAGTTATCCCTGCACACACA
**Sequencing Primer**	CAAACTTCCAAATGTGTGCA	AATGAATGCCCCTAATT
**Dispensation order**	CAGCTGCAGC	GATCACTATGAG
