# Supplementary material for: Assessing the fitness of a dual-antiviral drug resistant human influenza virus in the ferret model
Source: Commun Biol. 2022 Sep 28;5:1026. doi: 10.1038/s42003-022-04005-4 (PMC9517990; doi:10.1038/s42003-022-04005-4)
Supplement: Supplementary file 3 — Reporting Summary [file 42003_2022_4005_MOESM3_ESM.pdf]

nature portfolio

Corresponding author(s): Ian Barr

Last updated by author(s): Sep 9, 2022

Reporting Summary

Nature Portfolio wishes to improve the reproducibility of the work that we publish. This form provides structure for consistency and transparency in reporting. For further information on Nature Portfolio policies, see our Editorial Policies and the Editorial Policy Checklist.

Statistics

For all statistical analyses, confirm that the following items are present in the figure legend, table legend, main text, or Methods section.

n/a	Confirmed	
☐	☒	The exact sample size (n) for each experimental group/condition, given as a discrete number and unit of measurement
☐	☒	A statement on whether measurements were taken from distinct samples or whether the same sample was measured repeatedly
☐	☒	The statistical test(s) used AND whether they are one- or two-sided<br>*Only common tests should be described solely by name; describe more complex techniques in the Methods section.*
☒	☐	A description of all covariates tested
☐	☒	A description of any assumptions or corrections, such as tests of normality and adjustment for multiple comparisons
☐	☒	A full description of the statistical parameters including central tendency (e.g. means) or other basic estimates (e.g. regression coefficient) AND variation (e.g. standard deviation) or associated estimates of uncertainty (e.g. confidence intervals)
☐	☒	For null hypothesis testing, the test statistic (e.g. F, t, r) with confidence intervals, effect sizes, degrees of freedom and P value noted<br>*Give P values as exact values whenever suitable.*
☒	☐	For Bayesian analysis, information on the choice of priors and Markov chain Monte Carlo settings
☒	☐	For hierarchical and complex designs, identification of the appropriate level for tests and full reporting of outcomes
☒	☐	Estimates of effect sizes (e.g. Cohen's d, Pearson's r), indicating how they were calculated

Our web collection on statistics for biologists contains articles on many of the points above.

Software and code

Policy information about availability of computer code

Data collection	N/A
Data analysis	N/A

For manuscripts utilizing custom algorithms or software that are central to the research but not yet described in published literature, software must be made available to editors and reviewers. We strongly encourage code deposition in a community repository (e.g. GitHub). See the Nature Portfolio guidelines for submitting code & software for further information.

Data

Policy information about availability of data

All manuscripts must include a data availability statement. This statement should provide the following information, where applicable:

- Accession codes, unique identifiers, or web links for publicly available datasets
- A description of any restrictions on data availability
- For clinical datasets or third party data, please ensure that the statement adheres to our policy

All data generated or analysed during this study are included in this published article (and its supplementary information files). Numerical source data for Figure 1-5 has been made publically available in figshare with the identifier, https://doi.org/10.6084/m9.figshare.21008614 69. The whole genome sequence data of the clinically-derived viruses used in this study are available in GISAID with the identifiers A/South Korea/90207_d1/2020 (ID: 13655148), and A/South Korea/90207_d10/2020 (ID: 13655147).

Field-specific reporting

Please select the one below that is the best fit for your research. If you are not sure, read the appropriate sections before making your selection.

☒ Life sciences ☐ Behavioural & social sciences ☐ Ecological, evolutionary & environmental sciences

For a reference copy of the document with all sections, see nature.com/documents/nr-reporting-summary-flat.pdf

Life sciences study design

All studies must disclose on these points even when the disclosure is negative.

Sample size	In vivo, sample size of n=4 ferrets per group, limited because of the cost/housing/availability of ferrets, and the desire to reduce animal use but still ensure statistical significance analysis was possible. Previous ferret studies routinely use group sizes of 3-4 ferrets.
Data exclusions	No data were excluded from the analysis.
Replication	Findings in vivo were not replicated, due the cost/housing/availability of ferrets and animal ethics issues.
Randomization	Allocation of animals to groups was randomized.
Blinding	N/A

Reporting for specific materials, systems and methods

We require information from authors about some types of materials, experimental systems and methods used in many studies. Here, indicate whether each material, system or method listed is relevant to your study. If you are not sure if a list item applies to your research, read the appropriate section before selecting a response.

Materials & experimental systems

n/a	Involved in the study	
☐	☒	Antibodies
☐	☒	Eukaryotic cell lines
☒	☐	Palaeontology and archaeology
☐	☒	Animals and other organisms
☒	☐	Human research participants
☒	☐	Clinical data
☒	☐	Dual use research of concern

Methods

n/a	Involved in the study	
☒	☐	ChIP-seq
☒	☐	Flow cytometry
☒	☐	MRI-based neuroimaging

Antibodies

Antibodies used	Mouse anti-Influenza A nucleoprotein antibody MAB8251 (Millipore, USA), goat anti-mouse IgG-horse radish peroxidase (Biorad, US)
Validation	MAB8251 (LN3189197) certificate of analysis (https://www.merckmillipore.com/AU/en/product/Anti-Influenza-A-Antibody-nucleoprotein-clones-A1-A3-Blend,MM_NF-MAB8251?ReferrerURL=https%3A%2F%2Fwww.google.com%2F&bd=1#anchor_COA) and HRP-Ab certificate of analysis (https://commerce.bio-rad.com/prd/en/US/adirect/biorad?ts=1&cmd=InvoicePDFDisplay&fieldValue=1721011_64322730).

Eukaryotic cell lines

Policy information about cell lines

Cell line source(s)	MDCK CCL-34 (Canis familiaris, dog, epithelial, kidney): ATCC (USA)
Authentication	ATCC certificate of analysis could not be found.
Mycoplasma contamination	Mycoplasma negative.
Commonly misidentified lines (See ICLAC register)	N/A. MDCK cell lines are not listed on commonly misidentified cell line ICLAC register.

Animals and other organisms

Policy information about studies involving animals; ARRIVE guidelines recommended for reporting animal research

Laboratory animals	Outbred male and female ferrets (Mustela putorius furo), minimum of 12 weeks of age and 0.6 kg in body weight.
Wild animals	*Provide details on animals observed in or captured in the field; report species, sex and age where possible. Describe how animals were caught and transported and what happened to captive animals after the study (if killed, explain why and describe method; if released, say where and when) OR state that the study did not involve wild animals.*
Field-collected samples	*For laboratory work with field-collected samples, describe all relevant parameters such as housing, maintenance, temperature, photoperiod and end-of-experiment protocol OR state that the study did not involve samples collected from the field.*
Ethics oversight	University of Melbourne Animal Ethics Committee (project license no. 20033) in accordance with the Australian Government, National Health and Medical Research Council Australian code of practice for the care and use of animals for scientific purposes (8th edition).

Note that full information on the approval of the study protocol must also be provided in the manuscript.
